# Prevalence and socio-behavioral determinants of early childhood caries in children 1–5- year- old in Iran

**DOI:** 10.1371/journal.pone.0293428

**Published:** 2023-11-27

**Authors:** Ehsan Javadzadeh, Samaneh Razeghi, Ahmadreza Shamshiri, Hamid Heidarian Miri, Fatemeh Moghaddam, Robert J. Schroth, Simin Z. Mohebbi

**Affiliations:** 1 Research Center for Caries Prevention, Dentistry Research Institute, Tehran University of Medical Sciences, Tehran, Iran; 2 Community Oral Health Department, School of Dentistry, Tehran University of Medical Sciences, Tehran, Iran; 3 Infant Research center, University College Cork, Cork, Ireland; 4 Rady Faculty of Health Sciences, Department of Preventive Dental Sciences, Dr. Gerald Niznick College of Dentistry and Department of Pediatric and Child Health and Community Health Sciences, Max Rady, College of Medicine, Children’s Hospital Research Institute of University of Manitoba, Winnipeg, Canada; 5 Professor and Head of Community Oral Health Department, School of Dentistry, Tehran University of Medical Sciences, Tehran, Iran; Shahid Beheshti University of Medical Sciences School of Dentistry, ISLAMIC REPUBLIC OF IRAN

## Abstract

**Aim:**

According to the World Health Organization (WHO), early childhood caries (ECC) is still considered a major global health problem despite the general improvement in oral health practice. This study aimed to assess ECC’s prevalence, severity, and key social and behavioral determinants in Iranian children 1–5- years of age.

**Method:**

This cross-sectional study recruited Iranian 1–5-year-olds with a three-stage stratified clustered sampling method. After providing consent, parents were interviewed using a validated questionnaire, including questions on child characteristics and behavioral factors. Each child underwent a dental examination by one of four trained and calibrated dentists. Associations between key covariates of interest and primary outcome measures were assessed by multivariable logistic regression modeling and multivariate generalized negative binomial regression modeling after weightings were applied. Statistical analysis was performed using SPSS V25 and Stata V14.2 software packages. A p-value ≤ 0.05 was considered significant.

**Results:**

The mean age of 909 participants was 41.1±1.2 months, 48.6% were male, and 38.1% lived in rural areas. The overall prevalence of ECC was 53.2% and mean dmft score was 2.7± 0.3. Key determinants associated with ECC included living rurally (p = 0.009, OR = 2.13), consuming sweet drinks, sugary snacks, or both [p-value = 0.02 (OR = 2.53), and p-value<0.001 (OR = 4.96), respectively], and visible plaque (p<0.001, OR = 3.41). Covariates associated with dmft scores included residing in rural regions (p = 0.02, IRR = 1.31), having both sugary snacks and sweet drinks (p = 0.02, IRR = 1.85) compared to those had none, and visible dental plaque (p<0.001, IRR = 2.06).

**Conclusions:**

The prevalence of dental caries in children is high in Iran. The increase of ECC prevalence and severity from toddlers to preschoolers emphasizes on the critical importance of early interventions in toddlers. Improving access to care for rural children is essential along with the need to change dietary and self-care behaviors through multilevel efforts.

## Introduction

Early childhood caries (ECC) is the most common non-communicable disease in children and continues to remain as a serious global health challenge, both in developing and industrialized countries [1]. ECC is defined as the presence of any decay involving the primary dentition in children <72 months of age [2].

Despite improvements in the global prevalence of caries as a result of advancements in preventive dentistry the World Health Organization (WHO) still considers ECC to be a major health problem in most industrialized countries [3]. According to the results of the Global Burden of Disease Study in 2015, dental caries of primary teeth was the 12th most prevalent disease globally, involving more than 560 million children of all ages combined [4].

The prevalence of ECC is increasing rapidly in low- and middle-income countries, and dental caries is particularly prevalent among children living in economically deprived and disadvantaged communities [5,6]. Prevalence estimates for ECC have been reported to be between 1% and 12% in most developed countries, especially in immigrant populations, ethnic minorities or in rural areas [5,6], and is as high as 85% in disadvantaged groups in developing countries [7].

Since many ECC prevalence studies do not often include infants and toddlers < 2 years of age, the global understanding of the burden of caries in young children is limited. In Iran, studies conducted in children under age three are rare. A 2003 countrywide report for Iran revealed that the mean dmft had increased to 1.9 from 1.8 in 1998–99 [8,9]. The last published National Oral Health Survey reports for Iran in 2011–12 reported a mean dmft of 5.2 among children five- to six-year- of age [8].

Like many other non-communicable diseases (NCDs), both the etiology and prevention of ECC are strongly determined by socio-behavioral, economic, environmental, and societal factors, known as the social determinants of health [10]. As a multifactorial disease, ECC has numerous risk factors, including poverty, limited access to care, low parental education level, unsuitable lifestyle habits and diet, infant feeding practices (i.e., bottle feeding), frequent consumption of sugary drinks and foods, poor oral hygiene, and a lack of anticipatory guidance [11–13].

The strong influence of children and families’ health behaviors and practices along with socioeconomic pressures on the ECC development have made its etiology complex and multifactorial considering it as an explanation for inequalities that are increasing in low- and middle-income countries [1,14]. Societal and economic pressures often impact the health behaviors and practices of children and families and typically result in poor oral health [14].

The purpose of this study was to investigate the prevalence, severity, and key social and behavioral determinants of ECC in children 1-5- year of age in Iran.

## Methods

### Study design and sampling

This analytical cross-sectional survey was conducted among 12-71-month-old children and their parents from August 2020 to January 2021 in both rural and urban areas of four randomly selected provinces of Iran (out of 31). An equal-sized, multistage, stratified, cluster sampling approach was used to recruit the participants. As the first step for stratification, Iran’s provinces were categorized into four ordinal strata based on each province’s 5-6-year-old children’s average dmft score according to the 2012 Iranian National Oral Health Survey Report [15,16]. These cut-off points (≤5, 5.01–5.6, 5.61–6, >6) were determined in such a way that the total population of 5-6-year-old children in the provinces of each stratum is almost equal to the other strata. Next, from each stratum, a province was randomly selected through a lottery method, taking into account the necessity of geographic distribution. Randomly chosen representative provinces from four strata were Razavi Khorasan (4.48), West Azerbaijan (5.58), Yazd (5.96), and Qom (6.42).

The stratification was accomplished according to the location of residence (urban-rural), and available authorized sites for data collection (e.g., preschools, kindergartens, schools, health centers, mother and child health care and vaccination units, health houses (The Health House is the basic unit of the Iranian PHC (Primary Health Care) network to provide health services to rural population), gathering halls, cultural, religious, or occasional public sites, and even chiefs’ houses, especially in less privileged areas) respectively for secondary and tertiary sampling stages (Table 1).

**Table 1:** Survey multistage stratified cluster sampling approach details.

Sampling Stage	Strata	Cluster	Unit
**Primary**	All of Iran’s 31 provinces were categorized into four ordinal strata based on each province’s 5-6-year-old children’s average dmft score (≤5, 5.01–5.6, 5.61–6, >6)	Representative provinces were randomly selected out of each four strata including Razavi Khorasan, West Azerbaijan, Yazd, and Qom	Provinces
**Secondary**	Urban / Rural areas	Six cities including four provincial capitals and 15 villages were randomly chosen	Cities and Villages
**Tertiary**	Preschools, kindergartens, schools, health centers, mother and child health care and vaccination units, health houses, public sites, and gathering places, especially in less privileged areas	Randomly selected public sites and even all available ones in smaller cities, villages, and less privileged areas (as already mentioned) including a total of 55 final clusters (37 urban and 18 rural clusters)	Any public reference places (as previously mentioned) with more chance of sampling children from the study’s target age group according to inclusion/exclusion criteria
**Final**	12-71-month-old male / female children	Initially, 953 children were recruited into the survey including 466 males and 487 females	Individuals

### Clusters

Table 2 illustrates the distribution of participants in clusters (e.g., preschools, kindergartens, health centers, schools and other public sites) according to location of residence (urban and rural). Most of the participants were examined in kindergartens or preschools (n = 639).

**Table 2:** Distribution of participants in clusters according to location of residence (n = 909).

Location	Urban		Rural		Total	
	Clusters	Participants	Clusters	Participants	Clusters	Participants
**Preschools and Kindergartens**	32	540	2	91	34	631
**Health Centers or Houses**	2	5	3	54	5	59
**Schools**	0	0	1	10	1	10
**Other Public Sites**	3	18	11	171	14	189
**Homes**	0	0	1	20	1	20
**Total**	37	563	18	346	55	909

### Inclusion and exclusion criteria

The inclusion criteria were:

- Iranian nationality
- Children aged 12–71 months at the time of clinical oral examination
- Parents ability to understand the study as well as the questionnaire
- Parents’ willingness to sign the informed consent letter.

The exclusion criteria were:

- Children with non-Iranian nationalities
- Children in early mixed dentition; children who had any erupting permanent teeth just like first permanent molar
- Children whose parents failed to attend the day of the interview
- Children with major debilitating illnesses or disabilities, the presence of a significant health problem or a major systemic disease (Metabolic disorders such as Diabetes and Obesity, Respiratory diseases such as Asthma, Cystic Fibrosis, Gastrointestinal diseases, Neurologic diseases such as Cerebral Palsy and Rheumatoid Arthritis [17].
- Children who were uncooperative at the time of the dental examination.

Participating children were divided into five groups according to their ages (i.e., 12–23, 24–35, 36–47, 48–59, and 60–71 months of age). The children were later re-categorized as 12–47 (toddlers) and 48-71-month-old (preschoolers) for further analyses.

### Sample size calculation

The sample size was estimated according to the following formula in which *n* was the sample size, the *z*-score was set at 1.96 for the confidence level of 95%, *e* as the margin of error was set at 3.5%, and *N* as the target population size (Iranian children aged 12–71 months) was 7,032,347 according to Iran’s latest population census in 2016 [16].

Considering the results of a few similar available Iranian studies for the target age group, the overall expected prevalence (p) of ECC was set at 50% [15]. Accordingly, a theoretical sample size of 784 children was sought. To accommodate for the probable nonresponse of 20% of eligible participants due to the Covid-19 Pandemic, the sample size was increased to 950.

### Data collection and measurements

During each round of the survey at each site, the parents completed the questionnaire via interview followed by the child undergoing the oral examination.

#### Questionnaire

The study questionnaire was largely based on a previously used questionnaire [18,19]. The original questionnaire was in English had was translated into Persian. The study team revised some questions during translation to ensure that their intended meaning was preserved and to meet the study objectives.

The content validity of the questionnaire was assessed by a group of seven experts in community oral health and pediatric dentistry. Necessary modifications were performed to reach an agreement on controversial items. To ensure content accuracy, similarity, and clarity of the Persian version compared to the original questionnaire in English, two independent expert dental faculty members confirmed face validity.

Test-retest reliability was determined by pilot testing the questionnaire on separate days among a sample of 20 parents who were excluded from the final study sample. Neither the questions nor the answers were modified following this pilot study. The questionnaire included three parts as follow:

- demographic and socioeconomic background information: sex (male/female), child age, location of residence (Urban/Rural), Parents educational degree (Illiterate ‐ Reading & Writing ‐ Primary School / Middle School ‐ Diploma / Associate ‐ Bachelor / Master ‐ Ph.D. ‐ Above), and household’s self-reported economic status (Poor-Moderate / Good-Excellent),
- feeding practices and dietary habits: sweet snacking or drinking habits (None / Only Snack or Only Drink / Both), and number of meals per day (including both main meals and snacks),
- child’s oral health-related behaviors: toothbrushing frequency and fluoride toothpaste use.

### Clinical calibration and examination

Oral Examinations were conducted using the protocol for ECC diagnosis and risk assessment proposed by Evans et al. in 2018 [20]. ECC was defined following the AAPD definition [21,22] Examination equipment included disposable dental probes and mirrors, wooden single-use oral examination spatulas, and LED headlights. Four trained and calibrated examiners carried out oral examinations, each working with an assistant/data recorder. Younger children (up to 3 years of age) were examined using a knee-to-knee position while older children (over 3 years of age) were seated on a small chair during the dental examination.

Prior to data collection, the second author, a specialist in pediatric dentistry, was considered the gold standard during the calibration process. Subsequently, a calibration between the examiner dentists, the first author, and the specialist was performed by a sample of 50, 36-71-month-old preschool children (not included in the study population) as the pilot study. The examination was re-done one hour later to assess intra-examiner reliability. The calibration training and exercises were aimed and concentrated on the surface-by-surface accurate plaque level assessment, diagnosis of ECC, and differentiating ECC from other lesions with similar clinical appearances such as developmental defects, abnormalities, and/or opacities with non-carious nature such as diffuse opacities (e.g., deciduous molar hypomineralization), demarcated opacities, and enamel hypoplasia.

Calibration analysis involved the calculation of Kappa coefficients for ECC for both inter-examiner and intra-examiner agreements. The Kappa coefficients for the inter and intra-examiner reliability were set at κ ≥ 0.85 during both the pilot study and periodic (every 6 months) re-calibration sessions until the end of the study. As good inter and intra-examiner reliability agreements (κ ≥ 0.85) were achieved, the examiners were considered capable of conducting the epidemiological survey.

Plaque levels were assessed utilizing Silness & Löe plaque index (1964) [23], with codes ranging from 0 to 3 corresponding with the surface that had the highest index score. This was later dichotomized as visible/non-visible for further analysis. Following dental plaque assessment, plaque and debris were removed with sterile gauze, and dry the surface to provide better visualization to detect any existing caries lesions

The caries status of each surface of all erupted teeth was inspected, according to the protocol guidelines [20] to determine dmft (cumulative count of the number of decayed, missing due to caries, and filled primary tooth) scores.

Diagnostic findings by the examiners were directly recorded by an assistant into a printed chart sheet.

### Covid-19 pandemic considerations

The previously coordinated or authorized places for conducting interviews and oral examinations were visited and checked in terms of compliance with the protocols to minimize the chance of transmission or cross-contamination with Covid-19 agents.

Along with applying separation measures, appropriate zoning, and social distancing, sanitation measures including the use of new triple-layer surgical masks, disposable gloves, and alcohol hand rubs provided for free in waiting areas, were offered to participants. The appointments were set based on a schedule provided with the help of schools, preschools, kindergartens, health centers, and other public local administrators, staff, and even chiefs in less privileged areas to prevent crowding and manage social distancing as best as possible. Dental examinations were conducted in a single-participant, isolated, and well-ventilated room with a closed door and negative pressure relative to the surrounding area.

### Data handling and study variables

Questionnaire and oral examination data were entered into Microsoft Excel V2207 [24] and then converted to SPSS V25 [25] and Stata V14.2 [26] formats. Questionnaires and clinical data from the questionnaires were checked to be complete and without missing in the final statistical analysis. If case of missing data in some parts, we called the participants to complete the item by phone interview and if the person was not available, we excluded him/her from the study.

To investigate person-level ECC status, the presence of ECC was defined at the dmft ≥1 threshold, and ECC severity was defined by dmft score, both were considered as the survey’s dependent outcome variables.

Both the Silness & Löe plaque index and the presence of visible plaque were also considered as person-level oral hygiene indicators.

### Statistical analysis

The association between covariates of interest and the key dependent outcome measures (i.e., ECC, dmft score) and independent variables including Silness & Löe plaque index and visible plaque were investigated. Post-stratification survey weighting adjustments were performed to diminish inherent biases of the survey design and minimize any effects of challenges during data collection (e.g., overrepresented or underrepresented demographic groups) that may influence the results. Weights were calculated and applied to certain demographic variables including age, sex, and location so they could be compared with known characteristics of the general population of almost seven million Iranian children aged 12 to 71 months, obtained from Iran’s latest population census report in 2016 [16].

Descriptive statistics (i.e., frequencies, percentages, means ± standard deviations (SD) were performed. The association between ECC prevalence and study covariates were analyzed using bivariate (i.e., Chi Square analysis unadjusted Regression analysis). Those with p<0.2 were entered to multivariate analyses, using survey binary-response logistic regression modelling.

Association between the dmft score and study covariates were assessed using bivariate (i.e., ANOVA and unadjusted Regression analysis) and the independent variables with p<0.2 remained for multivariate association analyses, using survey generalized negative binomial regression modelling.

Besides the results from the survey’s regression analyses, the dataset was assessed for potential interactions through several subgroup analyses by age group, gender, and location utilizing the STATA software package version 14.2.

In the end, all obtained statistical results were exported to Microsoft Excel software Version 2207. P-values < 0.05 were considered significant.

### Ethics

Parents provided written informed consent in Persian prior to the children’s enrollment. Participation in the study was voluntary and each participant had the option to withdraw from the study at any stage of the study. All identifiable personal information was adequately disguised in the data in order to preserve the anonymity of the individuals involved.

Ethical clearance sought from Ethics Committee, Tehran University of Medical Sciences (Approval ID: IR.TUMS.DENTISTRY.REC.1398.070).

## Results

Overall, 96% of children underwent clinical examination, with 99% of those children having usable clinical data and were included in the analyses. A total of 909 children were included; 468 being toddlers and 441 preschoolers, 51.4% female, 61.9% living in urban areas, with a mean age of 41.12 (±1.2) months.

### Social and behavioral backgrounds

#### Socioeconomic factors

Socioeconomic status was assessed based on location of residence (urban-rural), parents’ educational degree, and self-reported household socioeconomic status (Table 3). The majority of fathers living in rural areas had completed "middle school/ or received a diploma" (46.1%). Similarly, the majority of mothers in rural areas possessed "middle school/ diploma" (58.4%). The education levels of participating parents appear in Table 3. While the majority of the urban households stated their socioeconomic status as “good to excellent” (52.4%), most of the rural households were considered to be “poor to moderate” (59.8%).

**Table 3:** Distribution (%) of children by socioeconomic status, feeding and dietary habits and oral hygiene behaviors (n = 909).

variables	Categories	Toddlers (12–47 months)						Preschoolers (48–71 months)						Total (12–71 months)					
		Urban		Rural		Total		Urban		Rural		Total		Urban		Rural		Total	
		Prevalence	SE	Prevalence	SE	Prevalence	SE	Prevalence	SE	Prevalence	SE	Prevalence	SE	Prevalence	SE	Prevalence	SE	Prevalence	SE
**Mother’s level of education**	Illiterate / or Primary School	1.5	0.9	36.2	6.2	11.8	2.2	4.6	2.3	29.2	4.4	11.8	2.6	2.7	1.2	33.5	4.9	11.8	2.0
	Middle School / Diploma	32.7	4.0	43.3	4.9	35.8	3.2	38.4	3.6	50.8	7.3	42.0	3.2	34.9	3.3	46.1	5.3	38.2	2.9
	Associate / Bachelor	40.2	3.7	19.8	2.8	34.2	2.7	30.5	2.5	18.8	5.7	27.1	2.4	36.5	2.9	19.4	2.5	31.5	2.2
	Master / Ph.D.	25.6	5.5	0.7	0.5	18.2	4.1	26.5	4.0	1.2	0.7	19.1	3.1	25.9	4.4	0.9	0.4	18.5	3.3
**Father’s level of education**	Illiterate / / Primary School	0.3	0.2	25.3	5.1	7.7	1.6	3.9	2.2	21.9	4.4	9.2	2.4	1.7	0.9	24.0	4.4	8.3	1.6
	Middle School / Diploma	22.0	4.8	55.7	4.4	32.0	3.7	31.3	5.2	62.8	4.7	40.6	4.0	25.6	4.4	58.4	2.6	35.3	3.5
	Associate / Bachelor	59.5	4.7	18.6	3.8	47.4	3.5	48.7	5.3	14.9	3.3	38.8	4.2	55.4	3.5	17.2	2.7	44.1	2.8
	Master / Ph.D.	18.1	4.4	0.3	0.3	12.9	3.2	16.0	2.1	0.4	0.4	11.4	1.6	17.3	2.9	0.3	0.2	12.3	2.2
**Self-declared Socioeconomic Status**	Poor–Moderate	49.9	5.9	56.1	4.5	51.8	4.3	43.9	7.9	65.9	6.4	50.4	5.8	47.6	6.1	59.8	4.4	51.2	4.5
	Good–Excellent	50.1	5.9	43.9	4.5	48.2	4.3	56.1	7.9	34.1	6.4	49.6	5.8	52.4	6.1	40.2	4.4	48.8	4.5
**Sugar intake**	No Sweet Drinks, No Sugary Snacks	10.9	2.8	11.4	2.8	11.1	2.1	3.5	1.2	15.1	3.1	6.9	1.4	8.1	2.1	12.8	2.0	9.5	1.6
	Sweet Drinks or Sugary Snacks	20.4	4.1	24.1	3.8	21.5	3.1	10.5	1.8	16.8	3.8	12.4	1.8	16.6	3.2	21.4	2.6	18.0	2.4
	Both Sweet Drinks and Sugary Snacks	68.7	4.9	64.4	3.9	67.4	3.6	86.0	2.2	68.1	3.5	80.7	2.3	75.3	4.0	65.8	3.2	72.5	3.0
**Meals per day**	Three Meals a Day or Less (< = 3)	31.8	4.3	44.3	7.1	35.5	3.5	41.4	3.0	39.8	6.7	41.0	2.9	35.5	3.2	42.6	5.3	37.6	2.7
	Four Meals a Day (= 4)	39.9	3.6	34.3	4.7	38.2	2.9	34.5	3.1	41.9	8.0	36.6	3.1	37.8	2.5	37.2	4.8	37.6	2.3
	Five Meals a Day or More (> = 5)	28.3	5.3	21.3	4.7	26.2	3.9	24.1	2.6	18.3	3.2	22.4	2.1	26.7	3.5	20.2	3.5	24.8	2.6
**Tooth brushing**	Never or Irregularly or Once a Week	53.9	4.3	80.8	3.9	61.8	3.4	23.7	3.1	71.0	4.7	37.6	3.5	42.3	3.7	77.0	3.6	52.6	3.2
	Several Times a Week (2 or 3)	19.0	3.1	8.6	3.4	15.9	2.5	30.9	3.2	16.2	2.9	26.5	2.5	23.5	2.6	11.5	2.0	20.0	2.0
	At Least Once a Day	27.1	3.9	10.6	3.6	22.2	2.9	45.4	3.4	12.9	3.0	35.9	3.1	34.1	3.1	11.5	3.0	27.4	2.5
**Use of Fluoridated Toothpaste**	Yes	31.7	3.5	16.3	3.0	27.1	2.6	62.2	2.9	19.4	3.0	49.6	3.0	43.4	3.0	17.5	2.4	35.7	2.5

SE: Standard Error, CI: Confidence Interval.

* Weighting was used to adjust the relative contribution of the respondents using known demographic characteristics including age, gender, and location of residence according to Iran’s latest population census in 2016.

#### Feeding & diet

Only 9.5% of children (11.1% of toddlers and 6.9% of preschoolers) had neither sugary snacks nor sweet drinks; 18% had consumed only one of the two items of sugary snacks or sweet drinks and the rest (72.5%) had both. The distribution of children according to feeding practices and dietary habits by age groups are summarized in Table 3. Considering the frequency of having meals (including main meals and snacks) during a day, 37.6% had three meals or less, 37.6% had four meals and 24.8% had five meals or more.

#### Child’s oral self-care

Parents reported that 27.4% of children (22.2% of toddlers and 35.9% of preschoolers) had their teeth brushed by parents at least once a day, 20% had their teeth brushed several times a week (2 or 3), while 52.6% had their teeth brushed weekly or less (Table 3). Only 35.7% of children (27.1% of toddlers and 49.6% of preschoolers) used fluoridated toothpaste.

#### Dental plaque

Overall, 49.0% of children had visible plaque. The plaque was visible on the dental surfaces of 38.6% of toddlers versus 66.0% of preschoolers. Prevalence of the Maximum Plaque Score on one- to five-year-old teeth surfaces using the Silness & Löe Plaque Index is shown in Table 4 (please see S.4 Table in S1 File for more details).

**Table 4:** Prevalence of the maximum plaque score on 1- to 5-year-olds teeth surfaces using the Silness & Löe plaque index (n = 909).

		No Detectable Plaque (Score = 0)					Detectable but Not Visible Plaque (Score = 1)					Visible Plaque (Score = 2)					Thick Plaque Layer (Score = 3)				
Age Group		Cases	Prevalence*	SE*	95 CI*		Cases	Prevalence*	SE*	95 CI*		Cases	Prevalence*	SE*	95 CI*		Cases	Prevalence*	SE*	95 CI*	
					Lower	Upper				Lower	Upper				Lower	Upper				Lower	Upper
**Toddlers**	**12–23 Months**	30	22.0	5.3	11.2	32.7	77	60.4	5.1	50.1	70.7	25	17.1	4.4	8.2	26.0	1	0.6	0.6	-0.6	1.8
	**24–35 Months**	9	6.6	2.3	1.9	11.3	66	45.1	4.1	36.8	53.5	62	41.7	4.0	33.7	49.8	11	6.5	2.6	1.3	11.8
	**36–47 Months**	23	10.7	2.6	5.4	16.0	68	38.4	5.2	27.9	48.8	83	43.5	5.9	31.8	55.3	13	7.4	2.7	2.0	12.9
	**Total**	62	13.2	2.4	8.3	18.1	211	48.2	3.0	42.2	54.2	170	33.8	3.5	26.9	40.8	25	4.8	1.3	2.1	7.4
**Preschoolers**	**48–59 Months**	17	7.4	2.6	2.1	12.7	60	27.0	3.5	19.8	34.1	125	53.7	4.0	45.7	61.7	25	11.9	3.0	5.8	18.1
	**60–71 Months**	10	5.6	1.8	1.9	9.3	64	28.3	4.8	18.5	38.0	118	59.1	5.3	48.4	69.9	22	7.0	2.6	1.8	12.2
	**Total**	27	6.5	1.9	2.8	10.3	124	27.6	3.4	20.8	34.4	243	56.4	3.7	49.0	63.8	47	9.5	2.4	4.7	14.3
**Total**		89	10.6	1.9	6.7	14.6	335	40.3	2.7	34.9	45.8	413	42.4	3.4	35.6	49.3	72	6.6	1.5	3.5	9.6

SE: Standard Error, CI: Confidence Interval.

* Weighting was used to adjust the relative contribution of the respondents using known demographic characteristics including age, gender, and location of residence according to Iran’s latest population census in 2016.

### Prevalence of ECC, dmft and dmfs

Overall, 53.2% of children had ECC; 35% in toddlers and 82.9% in preschoolers. The prevalence of ECC according to demographic characteristics is shown in Table 5. The mean dmft was 1.13 (±0.18) for toddlers; 0.34 (± 0.11) for 12 to 23-month-olds, 1.39 (±0.33) for 24 to 35-month-olds, and 1.71 (± 0.25) for 36 to 47-month-olds. The mean dmft was 5.29 (±0.32) for preschoolers; 4.53 (± 0.43) for 48 to 59-month-olds and 6.06 (± 0.37) for 60 to 71-month-olds. The mean dmft scores in age groups (toddler and preschooler) according to the location of residence (urban and rural) and gender (male and female) are illustrated in Table 6. The mean dmft score of the whole sample of 909 children was 2.72 (± 0.31). (Please see Table S.5 and S.6 for more details).

**Table 5:** Early Childhood Caries (ECC) prevalence in 1 to 5-year-olds in Iran (n = 909).

		Urban					Rural					Total				
Age Group		Cases	Prevalence*	SE*	95 CI*		Cases	Prevalence*	SE*	95 CI*		Cases	Prevalence*	SE*	95 CI*	
					Lower	Upper				Lower	Upper				Lower	Upper
**Toddlers**	**12–23 Months**	8	13.2	4.8	3.5	22.9	4	5.5	2.7	0.0	11.0	12	10.9	3.3	4.2	17.7
	**24–35 Months**	33	39.9	9.3	21.1	58.7	33	50.8	5.2	40.4	61.2	66	43.1	6.8	29.5	56.7
	**36–47 Months**	53	45.4	7.6	30.2	60.7	48	68.3	14.8	38.4	98.1	101	52.2	7.1	38.0	66.4
	**Total**	94	32.5	6.4	19.6	45.3	85	40.9	6.0	28.8	53.1	179	35.0	5.0	24.9	45.0
**Preschoolers**	**48–59 Months**	95	71.0	7.1	56.7	85.3	80	93.3	2.8	87.6	98.9	175	77.5	4.9	67.7	87.4
	**60–71 Months**	122	85.0	3.4	78.1	91.9	64	96.4	2.0	92.4	100.5	186	88.3	2.4	83.4	93.2
	**Total**	217	77.9	4.7	68.4	87.4	144	94.8	1.8	91.1	98.5	361	82.9	3.2	76.4	89.4
**Total**		311	49.8	7.0	35.7	64.0	229	61.4	4.7	52.0	70.8	540	53.2	5.2	42.7	63.7

SE: Standard Error, CI: Confidence Interval.

* Weighting was used to adjust the relative contribution of the respondents using known demographic characteristics including age, gender, and location of residence according to Iran’s latest population census in 2016.

**Table 6:** Mean dmft Score in 1- to 5-year-olds by age groups and location of residence in Iran (n = 909).

		Urban					Rural					Total				
Age Group		Count	Mean*	SE*	95% CI*		Count	Mean*	SE*	95% CI*		Count	Mean*	SE*	95% CI*	
					Lower	Upper				Lower	Upper				Lower	Upper
**Toddlers**	**12–23 Months**	77	0.37	0.15	0.07	0.67	56	0.27	0.14	0.00	0.54	133	0.34	0.11	0.12	0.57
	**24–35 Months**	81	1.27	0.45	0.37	2.17	67	1.68	0.30	1.07	2.29	148	1.39	0.33	0.73	2.06
	**36–47 Months**	121	1.21	0.16	0.89	1.53	66	2.88	0.68	1.52	4.25	187	1.71	0.25	1.21	2.21
	**Total**	279	0.94	0.21	0.52	1.36	189	1.59	0.29	0.99	2.18	468	1.13	0.18	0.77	1.49
**Preschoolers**	**48–59 Months**	138	4.11	0.60	2.91	5.31	89	5.53	0.41	4.70	6.35	227	4.53	0.43	3.67	5.39
	**60–71 Months**	146	5.86	0.45	4.96	6.76	68	6.55	0.73	5.08	8.02	214	6.06	0.37	5.32	6.81
	**Total**	284	4.98	0.45	4.08	5.88	157	6.03	0.31	5.42	6.65	441	5.29	0.32	4.65	5.92
**Total**		563	2.48	0.41	1.66	3.31	346	3.27	0.30	2.66	3.89	909	2.72	0.31	2.10	3.34

SE: Standard Error, CI: Confidence Interval.

* Weighting was used to adjust the relative contribution of the respondents using known populational characteristics including age, gender, and location of residence according to Iran’s latest population census in 2016.

The mean dmfs (decayed, missing, filled surfaces) score was 2.52 (± 0.35) for toddlers and 15.68 (± 1.02) for preschoolers. The total mean dmfs score (n = 909) was 7.54 (± 0.18).

### Analytical results

Before multivariate analysis, we entered all study independent variables into the regression model as bivariate analysis. Among all factors gender, father education and household’s self-reported socioeconomic status were not associated with ECC and dmft at all (P>0.2). Those with p value less than 0.2 were entered into the multivariate logistic regression model for final analysis.

Multivariable logistic regression modeling including age group, location of residence, mother education, frequencies of toothbrushing, use of fluoridated toothpaste, frequencies of sweet drinks and sugary snacks consumption, meals per day and visible dental plaque, with stepwise forward procedure retaining only significant factors revealed various factors to be associated with ECC occurrence (Table 7). Table 8 reveals the final results of association analysis with the dmft score by using multivariate generalized negative binomial regression model.

**Table 7:** Factors associating with ECC occurrence in 1- to 5-year-olds (n = 909) using multivariate logistic regression model.

Independent Variable	Value	OR*	SE*	95% CI*		P-value*
				Lower	Upper	
**Location of Residence**	Urban	1.00	‐‐‐	‐‐‐	‐‐‐	‐‐‐
	Rural	2.13	0.60	1.21	3.74	0.009
**Age Group**	12–23 months	1.00	‐‐‐	‐‐‐	‐‐‐	‐‐‐
	24–35 months	4.62	2.14	1.82	11.75	0.002
	36–47 months	6.98	2.62	3.28	14.85	<0.001
	48–59 months	20.63	8.28	9.20	46.28	<0.001
	60–71 months	56.71	20.79	27.13	118.54	<0.001
**Sweet Drinks and Sugary Snacks Consumption**	No Sweet Drinks, No Sugary Snacks	1.00	‐‐‐	‐‐‐	‐‐‐	‐‐‐
	Only Sweet Drinks or Only Sugary Snacks	2.53	1.01	1.14	5.63	0.02
	Both Sweet Drinks and Sugary Snacks	4.96	1.96	2.24	10.99	<0.001
**Meals per Day Including Main Meals and Snacks**	Three Meals a Day or Less (< = 3)	1.00	‐‐‐	‐‐‐	‐‐‐	‐‐‐
	Four Meals a Day (= 4)	2.31	0.48	1.52	3.52	<0.001
	Five Meals a Day or More (> = 5)	2.45	0.79	1.28	4.70	0.008
**Visible Dental Plaque by Naked Eyes**	No	1.00	‐‐‐	‐‐‐	‐‐‐	‐‐‐
	Yes	3.41	0.55	2.47	4.71	<0.001

SE: Standard Error, CI: Confidence Interval, OR: Odds Ratio.

* Weighting was used to adjust the relative contribution of the respondents using known populational characteristics including age, gender, and location of residence according to Iran’s latest population census in 2016.

**Table 8:** Factors associating with dmft score in 1- to 5-year-olds (n = 909) using multivariate generalized negative binomial regression model.

Independent Variable	Value	IRR*	SE*	95% CI*		P-value*
				Lower	Upper	
**Location of Residence**	Urban	1.00	‐‐‐	‐‐‐	‐‐‐	‐‐‐
	Rural	1.31	0.15	1.04	1.65	0.02
**Age Group**	12–23 months	1.00	‐‐‐	‐‐‐	‐‐‐	‐‐‐
	24–35 months	2.99	1.20	1.34	6.71	0.009
	36–47 months	3.49	1.19	1.75	6.94	0.001
	48–59 months	8.71	2.48	4.91	15.42	<0.001
	60–71 months	13.00	4.12	6.87	24.61	<0.001
**Sweet Drinks and Sugary Snacks Consumption**	No Sweet Drinks, No Sugary Snacks	1.00	‐‐‐	‐‐‐	‐‐‐	‐‐‐
	Only Sweet Drinks or Only Sugary Snacks	1.13	0.31	0.66	1.96	0.6
	Both Sweet Drinks and Sugary Snacks	1.85	0.47	1.11	3.09	0.02
**Meals per Day Including Main Meals and Snacks**	Three Meals a Day or Less (< = 3)	1.00	‐‐‐	‐‐‐	‐‐‐	‐‐‐
	Four Meals a Day (= 4)	1.35	0.15	1.08	1.68	0.009
	Five Meals a Day or More (> = 5)	1.28	0.23	0.89	1.84	0.2
**Visible Dental Plaque by Naked Eyes**	No	1.00	‐‐‐	‐‐‐	‐‐‐	‐‐‐
	Yes	2.06	0.27	1.58	2.69	<0.001

SE: Standard Error, CI: Confidence Interval, IRR: Incidence Rate Ratio.

* Weighting was used to adjust the relative contribution of the respondents using known populational characteristics including age, gender, and location of residence according to Iran’s latest population census in 2016.

As can be seen in Table 7, older age groups reveal more ECC occurrence. Among this study’s socio-economic factors, only location of residence (urban-rural) was found to be associated with ECC occurrence (p = 0.009, OR = 2.13). Having either sweet drinks or sugary snacks, or both, (compared to those who had none) revealed association with ECC [p-value = 0.02 (OR = 2.53), and p-value<0.001 (OR = 4.96), respectively]. Children who consumed four meals a day, and five meals a day or more, (compared to those who had three meals or less) were significantly more likely to have ECC [p-value <0.001 (OR = 2.31), and p-value = 0.008 (OR = 2.45), respectively]. Children who had visible dental plaque, (compared to those who had not), were more statistically more likely to have ECC (p<0.001, OR = 3.41).

Table 8 reveals that only location of residence (urban-rural) was found to be associated with dmft score (p = 0.02, IRR = 1.31). A significant association with higher dmft scores was found only among children who had both sugary snacks and sweet drinks (p = 0.02, IRR = 1.85) compared to those had none. and dmft score (p = 0.02, IRR = 1.31). Also, a significant association was found between number of meals and higher dmft score only among children who had four meals a day (p = 0.009, IRR = 1.35) compared to those had three meals or less. Association between visible dental plaque and higher dmft score was found significant (p<0.001, IRR = 2.06).

## Discussion

This study provides an important update on the prevalence and risk factors for ECC among Iranian children. Increased availability of ECC data will undoubtedly assist in the design and implementation of context-specific public oral health responses to control the disease. This will ultimately assist in identifying effective public health ECC control responses. Quite often those populations at greatest risk for ECC are rarely studied. Despite the clinical definition for ECC being more than 20 years old, some studies still do not classify ECC correctly, which hampers the ability to make comparisons with other studies [27]. In fact, only 45% of United Nations member countries have data on ECC using the accepted case definition by the American Academy of Pediatric Dentistry as the presence of any dmft in children younger than 6 years [27]. The present data supplements newly gathered national oral health data that did not include assessments of ECC in under-six-year-olds.

The total prevalence of ECC in this study was 53.2%, for the whole population aged 1–5- year- old. According to a recent systematic review aimed to describe the dental caries situation of preschool children living in 16 countries from six continents, the prevalence of ECC ranged from 22.5% in India to 90% in Indonesia [27]. Another systematic review, showed a combined prevalence of 48% [95% CI 43, 53], with variations both between and within countries [28]. The overall prevalence of ECC in the United States of America from 2013 to 2018 was 18.6 percent [29]. The challenge in making comparisons between studies is that different studies include children of different infant and preschool ages and some use different diagnostic criteria for caries. The high prevalence of ECC can be due to several reasons such as lack of oral hygiene practices, high cost of dental treatment, and limited accessibility of dental services, and the lack of knowledge about the importance of primary dentition [30–36].

In this present study, the prevalence of ECC in toddlers was 35% ranging from 10 to 52% in the three age groups. In 2005, the ECC prevalence in 1 to 3-year-olds was reported at 3% to 33% by Mohebbi’s cross-sectional study for the children living in Tehran [11]. Nevertheless, the present data was gathered throughout the whole country versus the previous data, which was related to Tehran, the capital city of Iran. In general, more international variation in ECC prevalence exists for those aged two years and older but not for the younger ones [11]. In this study, there is a great increase in ECC prevalence comparing 12 to 23-month-olds with the next age group, 24 to 35-month-olds, from about 10 percent to about 40 percent. Such an increase is not seen in comparison to the other age groups. This sudden increase in the prevalence of the disease between two age groups emphasizes the importance of intervention at the maximum age of one year to prevent the occurrence of dental caries in young children, which is in line with the international guidelines of AAPD and European Academy of Padiatric Dentistry[13,37].

The prevalence of ECC in preschoolers was found to be 83%. Consistent with previous surveys [38,39], we found a significant association between the child’s age and the ECC prevalence. With the increase in age, the duration of exposure of teeth to cariogenic factors increases. Therefore, it is rational to yield higher prevalence in older children. Previous surveys conducted in different provinces of Iran have reported various prevalence values in 3-5-year-old children, as 55% in Shiraz [40], 51.2% in Rafsanjan [41], 73.2% in Babol [42] and 68.1% in Qazvin [43]. Our figure is higher than the previous reports, which follows the increasing pattern of ECC and indicates a lack of proper oral health care for children in this age group.

The mean dmft score of the whole sample of 909 children was found to be 2.72 while it was 1.13 in toddlers and 5.29 in preschoolers. The two previous most recent national surveys report the mean dmft score in 5–6-year-old children to be 1.9 in 2003–4 and 5.16 in 2011–12 [15,44]. Our five-year olds’ dmft of 6.06 reveals an increase in the severity of ECC in comparison to older data, which was originally high enough. The increase in dmft when comparing 12 to 23-month-olds with the next age group, 24- to 35-month-olds, from about 0.3 to about 1.5 accounts for 5 times more severity of the disease. The slope of caries increase between these two young age groups is much higher than that of other age groups emphasizing the critical age of one year of age for the implementation of preventive programs in the health care system. On the other hand, the COVID-19 pandemic (the present study was conducted during Covid-19 pandemic) has a significant influence on patients’ attendance to pediatric dental visits for prevention or treatment demands which may yield increased number of oral health-related complications in the long run [45]. The increase in dmft scores compared with past national estimates may be a result of limited access to care resulting from the COVID-19 pandemic and the closure of dental offices. Global inroads into early childhood oral health prevention were dealt significant setbacks during COVID-19 as emphasis was placed on emergent and urgent care and children’s preventive services for all, particularly high-risk populations, were considered unnecessary.

In this present study, the caries experience was comparable in boys and girls, being slightly more (insignificant) in boys similar to that reported by Peressini, Mulchandani and Evan Tusek [46,47]. However, Jianbo et al. found that the mean dmft was higher in female Chinese children [48]. A recent Canadian study revealed that there are sex-based differences in the oral microbiome and mycobiome between boys and girls, which might influence caries risk [49]. No gender discrimination in health outcomes among the present children might be considered a desirable achievement. Nevertheless, the children revealed high prevalence and severity of ECC regardless of their gender which emphasizes on overall child neglect in oral health aspects.

Global evidence confirms that socioeconomic status is a risk factor for ECC [50–52]. Among study socio-economic associating factors, only location of residence was significantly associated with ECC occurrence and dmft score. Higher ECC occurrence and dmft score was found in children who live in rural areas which is similar to other studies’ findings [48,53,54]. Probably due to a variety of reasons, including reduced access to care because a lack of dental practitioners for children, and potential limited oral health knowledge and low parental awareness of the importance of early childhood oral health care [55].

Sugar intake is currently recognized as the most closely related dietary factor in dental caries. The World Health Organization (WHO) recommends to limit sugar intake to less than 10% of total energy intake for both adults and children in the Sugar Intake Guidelines published in 2015 [56,57]. A systematic review showed that ECC is related to frequent sweets consumption [58]. In particular, numerous studies have proven that sweets play a major role in the occurrence and development of ECC [59–61]. In the present study, frequency consumption of sweets was prevalent as about 90.5% of the children had only sweet drinks/sugary snacks or both in between their meals. The prevalence of ECC among these children was higher in comparison to those who had none. Findings from this current study are consistent with previous reports of an association between prolonged high sugar consumption between meals and ECC in children [62,63]. Also, a significant association with higher dmft score have been found only among children who had both sugary snacks and sweet drinks compared to those had none accordance with other studies [64,65].

In the present study more ECC prevalence was observed in both groups of children who had four meals a day and five meals a day or more, (compared to those who had three main meals or less). This finding was contrary to which reported by Sanaa N. Al-Haj Ali et al. have been found no any association between frequencies of main meals and higher risk of ECC. This might be attributed to some extent to other dietary habits and culture [66].

A significant association was reported in this present study between the number of meals and higher dmft scores. Specifically, children who consumed four meals a day compared to those had three meals or less had higher dmft scores. For children under the age of 5 years, frequent intake may be necessary due to small appetites, but more than six periods of eating/drinking per day is considered as dietary risk factor to occurrence of ECC [67]. Biochemical changes in the oral cavity, such as the metabolism of sugars from food to acids which decreases pH of the environment leading to enamel demineralization and formation of carious lesion. So, the more frequently the dental plaque pH drops below a critical point, the more frequently demineralization occurs.

In present study, only 27.4% of children brushed their teeth at least once a day, which is quite low when compared to 52.6% of children with never/irregularly or once a week, and 20% of children with several times a week toothbrushing. While our results suggest that brushing frequency was not significantly associated with ECC and higher dmft scores, this is an area for health promotion so that children receive exposure to fluoridated toothpaste to protect primary teeth and reduce plaque levels, which contribute to increased caries risk. Our findings are in contrast to the results from a meta-analysis revealing the prevalence of ECC to be higher in children with no/irregular brushing [68]. However, some others reported that the brushing frequency was not significantly related to caries development [69,70].

According to Pieper et al., children who received active help from their parents for toothbrushing after 3 years of age had a significantly lower dmft score than children whose parents did not supervise their brushing beyond this age [71]. This might be attributed to inadequate toothbrushing either due to lack of parents’ awareness of the importance of their active engagement in brushing their children’s teeth in this age group or their failure to supervise their children’s uncooperative behavior who might be resistant to opening their mouths while brushing their teeth [72]. While lack of toothbrushing was a risk factor for ECC [32], irregular brushing at 18 months of age was a highly significant predictor of developing ECC [73]. About 75 percent of children in the present study had irregular toothbrushing and moreover the quality of brushing was unclear to us. This might lead to an overall insufficient oral hygiene and high ECC occurrence regardless of frequency of brushing. Therefore, professionals should give parents special attention and assist in improving and optimizing their toothbrushing behavior during children’s preschool years.

Furthermore, the use of fluoride toothpaste is generally described accepted tool to bring fluoride into the oral cavity [74] and an effective prevention method of ECC occurrence [13,75]. In our study, only 35.7% of parents used fluoride toothpaste for their children. However, as the same results of Boustedt et al. no significant relationship existed between fluoride toothpaste usage and ECC risk [74]. In fact, the caries-preventive effect is statistically significant only for concentration of 1000 ppm and above [13]; moreover, wide irregular tooth brushing by the present young population may compromise the effect of fluoride.

Previous studies found that visible dental plaque is an important predictor of new carious lesions [51,76,77]. In our study, visible dental plaque had a significant relationship with ECC and higher dmft score. In another study that was conducted in Iran the clear relationships between the presence of ECC and dental plaque was verified in toddlers [11,42].

### Study limitation and strengths

Although the present findings are important, the results must be interpreted in light of the study’s limitations. First, using the WHO criteria of caries detection without bitewing radiographs and with inherent limitations of dmf index may underestimate the actual prevalence of dental caries. Second, there may be difficulty in getting accurate information from parents because it is based on the power of recall. Response bias is also likely.

Severity was just measured by mean dmf score in this study while it may be considered a subjective indicator too. Finally, dental service utilization might affect children’s oral health which might be considered in future studies.

The strengths of this survey include First, a representative sample which was ensured by an equal-sized, stratified, multistage random sampling approach, the use of a reliable index, and the application of statistical analysis models appropriate to the multilevel nature of the sampling with a weighted process which made the results be close to reality and permit us to generalize the findings to the population and second, there were no missing values in the outcome variables lack of information on the history of visiting a dentist, severity is a subjective indicator which does not seem to be evaluated in this study as no forms of patient or proxy-reported outcome used, and the inherent limitations dmft are not discussed.

### Conclusion

The prevalence of ECC in Iranian children is high. The increase in ECC prevalence and severity over past national estimates is concerning. The increase from one year of old to two years of old emphasizes on the critical importance of early preventive interventions during the first year of life, including the first dental visit no later than 12 months of age. Special attention must be given to children from rural areas and parents and communities must continue to be informed about the role of dietary and self-care behaviors in caries-risk.

## Supporting information

S1 Checklist STROBE-checklist-v4-combined-PlosMedicine. STROBE-checklist-v4-combined-PlosMedicine.
(DOCX)

S1 File Table S.4. Prevalence of the maximum plaque score on 1- to 5-year-olds teeth surfaces using the Silness & Löe plaque index (n = 909). Table S.5. Early Childhood Caries (ECC) Prevalence in 1 to 5-year-olds in Iran (n = 909). Table S.6. Mean dmft Score in 1- to 5-year-olds by demographic characteristics in Iran (n = 909).
(DOCX)

S1 Data (XLSX)
