# Supplementary material for: Prevalence and socio-behavioral determinants of early childhood caries in children 1–5- year- old in Iran
Source: PLoS One. 2023 Nov 27;18(11):e0293428. doi: 10.1371/journal.pone.0293428 (PMC10681183; doi:10.1371/journal.pone.0293428)
Supplement: S1 File — Table S.4. Prevalence of the maximum plaque score on 1- to 5-year-olds teeth surfaces using the Silness & Löe plaque index (n = 909). Table S.5. Early Childhood Caries (ECC) Prevalence in 1 to 5-year-olds in Iran (n = 909). Table S.6. Mean dmft Score in 1- to 5-year-olds by demographic characteristics in Iran (n = 909). (DOCX) [file pone.0293428.s002.docx]

| **Table S.4. Prevalence of the maximum plaque score on 1- to 5-year-olds teeth surfaces using the Silness & Löe plaque index (n=909).** | | | | | | | | | | | | | | | | | | | | | | |
| --- | --- | --- | --- | --- | --- | --- | --- | --- | --- | --- | --- | --- | --- | --- | --- | --- | --- | --- | --- | --- | --- | --- |
|  | | | **No Detectable Plaque**  **(Score = 0)** | | | | | **Detectable but Not Visible Plaque**  **(Score = 1)** | | | | | **Visible Plaque**  **(Score = 2)** | | | | | **Thick Plaque Layer**  **(Score = 3)** | | | | |
| **Age Group** | | Gender | Cases | Prevalence* | SE* | 95 CI* | | Cases | Prevalence* | SE* | 95 CI* | | Cases | Prevalence* | SE* | 95 CI* | | Cases | Prevalence* | SE* | 95 CI* | |
|  |  |  |  |  |  | Lower | Upper |  |  |  | Lower | Upper |  |  |  | Lower | Upper |  |  |  | Lower | Upper |
| Toddler | **12 - 23 months** | Female | 15 | 24.2 | 5.7 | 12.8 | 35.6 | 34 | 56.8 | 7.9 | 40.9 | 72.7 | 14 | 19.0 | 5.7 | 7.6 | 30.4 | 0 | 0.0 | 0.0 | 0.0 | 0.0 |
|  |  | Male | 15 | 19.9 | 6.9 | 6.1 | 33.7 | 43 | 63.7 | 5.8 | 52.2 | 75.3 | 11 | 15.2 | 4.8 | 5.7 | 24.8 | 1 | 1.1 | 1.2 | -1.2 | 3.5 |
|  |  | Total | 30 | 22.0 | 5.3 | 11.2 | 32.7 | 77 | 60.4 | 5.1 | 50.1 | 70.7 | 25 | 17.1 | 4.4 | 8.2 | 26.0 | 1 | 0.6 | 0.6 | -0.6 | 1.8 |
|  | **24 - 35 months** | Female | 7 | 11.6 | 4.6 | 2.3 | 20.9 | 36 | 34.5 | 7.2 | 20.0 | 49.1 | 34 | 48.3 | 6.4 | 35.5 | 61.1 | 7 | 5.6 | 2.3 | 0.8 | 10.3 |
|  |  | Male | 2 | 1.9 | 1.4 | -0.8 | 4.6 | 30 | 55.1 | 5.9 | 43.3 | 66.9 | 28 | 35.6 | 6.0 | 23.5 | 47.6 | 4 | 7.4 | 4.1 | -0.9 | 15.8 |
|  |  | Total | 9 | 6.6 | 2.3 | 1.9 | 11.3 | 66 | 45.1 | 4.1 | 36.8 | 53.5 | 62 | 41.7 | 4.0 | 33.7 | 49.8 | 11 | 6.5 | 2.6 | 1.3 | 11.8 |
|  | **36 - 47 months** | Female | 14 | 11.1 | 3.6 | 3.9 | 18.3 | 38 | 39.8 | 5.9 | 28.0 | 51.6 | 48 | 44.3 | 6.6 | 31.0 | 57.6 | 5 | 4.9 | 2.9 | -1.0 | 10.7 |
|  |  | Male | 9 | 10.3 | 3.7 | 2.8 | 17.8 | 30 | 37.0 | 5.4 | 26.1 | 47.9 | 35 | 42.8 | 7.2 | 28.3 | 57.4 | 8 | 9.9 | 3.3 | 3.2 | 16.6 |
|  |  | Total | 23 | 10.7 | 2.6 | 5.4 | 16.0 | 68 | 38.4 | 5.2 | 27.9 | 48.8 | 83 | 43.5 | 5.9 | 31.8 | 55.3 | 13 | 7.4 | 2.7 | 2.0 | 12.9 |
|  | **Total** | Female | 36 | 15.7 | 3.3 | 9.2 | 22.3 | 108 | 43.9 | 4.3 | 35.2 | 52.5 | 96 | 36.9 | 4.5 | 27.9 | 46.0 | 12 | 3.4 | 1.3 | 0.8 | 6.0 |
|  |  | Male | 26 | 10.8 | 2.7 | 5.3 | 16.3 | 103 | 52.3 | 3.5 | 45.3 | 59.3 | 74 | 30.9 | 4.1 | 22.5 | 39.2 | 13 | 6.1 | 1.9 | 2.3 | 9.8 |
|  |  | Total | 62 | 13.2 | 2.4 | 8.3 | 18.1 | 211 | 48.2 | 3.0 | 42.2 | 54.2 | 170 | 33.8 | 3.5 | 26.9 | 40.8 | 25 | 4.8 | 1.3 | 2.1 | 7.4 |
| Preschooler | **48 - 59 months** | Female | 12 | 12.8 | 5.2 | 2.3 | 23.3 | 31 | 28.6 | 6.4 | 15.6 | 41.5 | 54 | 48.3 | 8.0 | 32.3 | 64.3 | 12 | 10.3 | 3.8 | 2.7 | 18.0 |
|  |  | Male | 5 | 2.3 | 1.2 | -0.1 | 4.7 | 29 | 25.4 | 3.2 | 18.9 | 32.0 | 71 | 58.8 | 5.0 | 48.8 | 68.8 | 13 | 13.4 | 5.3 | 2.8 | 24.1 |
|  |  | Total | 17 | 7.4 | 2.6 | 2.1 | 12.7 | 60 | 27.0 | 3.5 | 19.8 | 34.1 | 125 | 53.7 | 4.0 | 45.7 | 61.7 | 25 | 11.9 | 3.0 | 5.8 | 18.1 |
|  | **60 - 71 months** | Female | 5 | 7.3 | 3.2 | 0.8 | 13.8 | 31 | 28.6 | 7.0 | 14.5 | 42.7 | 64 | 61.3 | 7.8 | 45.7 | 77.0 | 6 | 2.7 | 1.5 | -0.3 | 5.8 |
|  |  | Male | 5 | 4.0 | 1.6 | 0.7 | 7.3 | 33 | 27.9 | 6.1 | 15.7 | 40.1 | 54 | 57.0 | 4.8 | 47.3 | 66.8 | 16 | 11.1 | 4.2 | 2.6 | 19.5 |
|  |  | Total | 10 | 5.6 | 1.8 | 1.9 | 9.3 | 64 | 28.3 | 4.8 | 18.5 | 38.0 | 118 | 59.1 | 5.3 | 48.4 | 69.9 | 22 | 7.0 | 2.6 | 1.8 | 12.2 |
|  | **Total** | Female | 17 | 10.1 | 3.3 | 3.5 | 16.7 | 62 | 28.6 | 5.1 | 18.3 | 38.9 | 118 | 54.8 | 7.0 | 40.7 | 68.8 | 18 | 6.6 | 2.1 | 2.3 | 10.9 |
|  |  | Male | 10 | 3.1 | 1.2 | 0.8 | 5.5 | 62 | 26.6 | 3.7 | 19.2 | 34.1 | 125 | 57.9 | 2.5 | 52.9 | 63.0 | 29 | 12.3 | 3.9 | 4.5 | 20.1 |
|  |  | Total | 27 | 6.5 | 1.9 | 2.8 | 10.3 | 124 | 27.6 | 3.4 | 20.8 | 34.4 | 243 | 56.4 | 3.7 | 49.0 | 63.8 | 47 | 9.5 | 2.4 | 4.7 | 14.3 |
| **Total** | | Female | 53 | 13.6 | 2.9 | 7.8 | 19.4 | 170 | 38.0 | 3.6 | 30.8 | 45.3 | 214 | 43.7 | 4.9 | 33.9 | 53.6 | 30 | 4.6 | 1.3 | 2.1 | 7.2 |
|  |  | Male | 36 | 7.9 | 1.9 | 4.1 | 11.7 | 165 | 42.5 | 3.3 | 35.9 | 49.2 | 199 | 41.2 | 3.4 | 34.4 | 47.9 | 42 | 8.4 | 2.3 | 3.9 | 13.0 |
|  |  | Total | 89 | 10.6 | 1.9 | 6.7 | 14.6 | 335 | 40.3 | 2.7 | 34.9 | 45.8 | 413 | 42.4 | 3.4 | 35.6 | 49.3 | 72 | 6.6 | 1.5 | 3.5 | 9.6 |
| SE: Standard Error, CI: Confidence Interval  * Weighting was used to adjust the relative contribution of the respondents using known demographic characteristics including age, gender, and location of residence according to Iran's latest population census in 2016. | | | | | | | | | | | | | | | | | | | | | | |

| **Table S.5. Early Childhood Caries (ECC) Prevalence in 1 to 5-year-olds in Iran (n=909).** | | | | | | | | | | | | | | | | | |
| --- | --- | --- | --- | --- | --- | --- | --- | --- | --- | --- | --- | --- | --- | --- | --- | --- | --- |
|  | | | **Urban** | | | | | **Rural** | | | | | **Total** | | | | |
| **Age Group** | | Gender | Cases | Prevalence* | SE* | 95 CI* | | Cases | Prevalence* | SE* | 95 CI* | | Cases | Prevalence* | SE* | 95 CI* | |
|  |  |  |  |  |  | Lower | Upper |  |  |  | Lower | Upper |  |  |  | Lower | Upper |
| Toddler | **12 - 23 months** | Female | 2 | 7.7 | 5.8 | -4.0 | 19.4 | 3 | 7.0 | 4.2 | -1.5 | 15.4 | 5 | 7.5 | 4.3 | -1.2 | 16.1 |
|  |  | Male | 6 | 18.4 | 8.1 | 2.1 | 34.6 | 1 | 4.2 | 4.2 | -4.2 | 12.6 | 7 | 14.2 | 5.5 | 3.0 | 25.3 |
|  |  | Total | 8 | 13.2 | 4.8 | 3.5 | 22.9 | 4 | 5.5 | 2.7 | 0.0 | 11.0 | 12 | 10.9 | 3.3 | 4.2 | 17.7 |
|  | **24 - 35 months** | Female | 21 | 41.2 | 10.7 | 19.7 | 62.7 | 19 | 51.1 | 8.6 | 33.9 | 68.3 | 40 | 44.1 | 8.0 | 28.1 | 60.1 |
|  |  | Male | 12 | 38.7 | 9.5 | 19.6 | 57.7 | 14 | 50.5 | 8.0 | 34.4 | 66.7 | 26 | 42.2 | 7.1 | 28.0 | 56.4 |
|  |  | Total | 33 | 39.9 | 9.3 | 21.1 | 58.7 | 33 | 50.8 | 5.2 | 40.4 | 61.2 | 66 | 43.1 | 6.8 | 29.5 | 56.7 |
|  | **36 - 47 months** | Female | 29 | 44.9 | 5.6 | 33.5 | 56.2 | 30 | 72.4 | 15.8 | 40.6 | 104.3 | 59 | 53.0 | 6.2 | 40.5 | 65.6 |
|  |  | Male | 24 | 46.0 | 13.6 | 18.6 | 73.3 | 18 | 64.3 | 15.1 | 33.9 | 94.8 | 42 | 51.5 | 10.7 | 30.0 | 72.9 |
|  |  | Total | 53 | 45.4 | 7.6 | 30.2 | 60.7 | 48 | 68.3 | 14.8 | 38.4 | 98.1 | 101 | 52.2 | 7.1 | 38.0 | 66.4 |
|  | **Total** | Female | 52 | 30.8 | 6.6 | 17.5 | 44.2 | 52 | 43.0 | 8.8 | 25.2 | 60.7 | 104 | 34.4 | 5.5 | 23.3 | 45.5 |
|  |  | Male | 42 | 34.0 | 7.7 | 18.5 | 49.5 | 33 | 39.1 | 5.2 | 28.6 | 49.5 | 75 | 35.5 | 5.8 | 23.9 | 47.1 |
|  |  | Total | 94 | 32.5 | 6.4 | 19.6 | 45.3 | 85 | 40.9 | 6.0 | 28.8 | 53.1 | 179 | 35.0 | 5.0 | 24.9 | 45.0 |
| Preschooler | **48 - 59 months** | Female | 47 | 71.1 | 8.9 | 53.3 | 89.0 | 36 | 93.3 | 3.9 | 85.5 | 101.1 | 83 | 77.6 | 6.0 | 65.6 | 89.7 |
|  |  | Male | 48 | 70.8 | 6.6 | 57.4 | 84.1 | 44 | 93.2 | 3.2 | 86.7 | 99.7 | 92 | 77.4 | 4.9 | 67.5 | 87.3 |
|  |  | Total | 95 | 71.0 | 7.1 | 56.7 | 85.3 | 80 | 93.3 | 2.8 | 87.6 | 98.9 | 175 | 77.5 | 4.9 | 67.7 | 87.4 |
|  | **60 - 71 months** | Female | 56 | 80.4 | 6.9 | 66.5 | 94.4 | 34 | 96.5 | 2.1 | 92.3 | 100.8 | 90 | 85.2 | 4.7 | 75.6 | 94.7 |
|  |  | Male | 66 | 89.3 | 4.0 | 81.1 | 97.4 | 30 | 96.3 | 2.6 | 91.0 | 101.7 | 96 | 91.3 | 3.0 | 85.4 | 97.3 |
|  |  | Total | 122 | 85.0 | 3.4 | 78.1 | 91.9 | 64 | 96.4 | 2.0 | 92.4 | 100.5 | 186 | 88.3 | 2.4 | 83.4 | 93.2 |
|  | **Total** | Female | 103 | 75.7 | 7.2 | 61.2 | 90.3 | 70 | 94.9 | 2.7 | 89.6 | 100.3 | 173 | 81.4 | 4.9 | 71.6 | 91.1 |
|  |  | Male | 114 | 79.9 | 4.1 | 71.8 | 88.1 | 74 | 94.7 | 2.0 | 90.7 | 98.7 | 188 | 84.3 | 3.0 | 78.2 | 90.4 |
|  |  | Total | 217 | 77.9 | 4.7 | 68.4 | 87.4 | 144 | 94.8 | 1.8 | 91.1 | 98.5 | 361 | 82.9 | 3.2 | 76.4 | 89.4 |
| **Total** | | Female | 155 | 48.0 | 7.3 | 33.4 | 62.6 | 122 | 62.7 | 7.0 | 48.7 | 76.8 | 277 | 52.3 | 5.6 | 41.1 | 63.6 |
|  |  | Male | 156 | 51.5 | 8.4 | 34.7 | 68.3 | 107 | 60.1 | 5.4 | 49.2 | 71.1 | 263 | 54.1 | 6.2 | 41.6 | 66.6 |
|  |  | Total | 311 | 49.8 | 7.0 | 35.7 | 64.0 | 229 | 61.4 | 4.7 | 52.0 | 70.8 | 540 | 53.2 | 5.2 | 42.7 | 63.7 |
| SE: Standard Error, CI: Confidence Interval  * Weighting was used to adjust the relative contribution of the respondents using known demographic characteristics including age, gender, and location of residence according to Iran's latest population census in 2016. | | | | | | | | | | | | | | | | | |

| **Table S.6. Mean dmft Score in 1- to 5-year-olds by demographic characteristics in Iran (n=909).** | | | | | | | | | | | | | | | | | |
| --- | --- | --- | --- | --- | --- | --- | --- | --- | --- | --- | --- | --- | --- | --- | --- | --- | --- |
|  | | | **Urban** | | | | | **Rural** | | | | | **Total** | | | | |
| **Age Group** | | Gender | Count | Mean* | SE* | 95% CI* | | Count | Mean* | SE* | 95% CI* | | Count | Mean* | SE* | 95% CI* | |
|  |  |  |  |  |  | Lower | Upper |  |  |  | Lower | Upper |  |  |  | Lower | Upper |
| Toddler | **12 - 23 months** | Female | 34 | 0.35 | 0.26 | -0.18 | 0.87 | 29 | 0.34 | 0.22 | -0.11 | 0.79 | 63 | 0.34 | 0.19 | -0.05 | 0.73 |
|  |  | Male | 43 | 0.40 | 0.18 | 0.03 | 0.77 | 27 | 0.21 | 0.21 | -0.21 | 0.63 | 70 | 0.34 | 0.14 | 0.06 | 0.62 |
|  |  | Total | 77 | 0.37 | 0.15 | 0.07 | 0.67 | 56 | 0.27 | 0.14 | 0.00 | 0.54 | 133 | 0.34 | 0.11 | 0.12 | 0.57 |
|  | **24 - 35 months** | Female | 44 | 1.39 | 0.51 | 0.37 | 2.41 | 40 | 1.80 | 0.46 | 0.86 | 2.73 | 84 | 1.51 | 0.39 | 0.73 | 2.29 |
|  |  | Male | 37 | 1.15 | 0.43 | 0.29 | 2.02 | 27 | 1.57 | 0.35 | 0.88 | 2.27 | 64 | 1.28 | 0.32 | 0.64 | 1.92 |
|  |  | Total | 81 | 1.27 | 0.45 | 0.37 | 2.17 | 67 | 1.68 | 0.30 | 1.07 | 2.29 | 148 | 1.39 | 0.33 | 0.73 | 2.06 |
|  | **36 - 47 months** | Female | 66 | 1.22 | 0.28 | 0.67 | 1.77 | 39 | 2.56 | 0.70 | 1.14 | 3.97 | 105 | 1.62 | 0.31 | 1.00 | 2.24 |
|  |  | Male | 55 | 1.20 | 0.21 | 0.79 | 1.62 | 27 | 3.19 | 0.97 | 1.25 | 5.13 | 82 | 1.80 | 0.35 | 1.10 | 2.49 |
|  |  | Total | 121 | 1.21 | 0.16 | 0.89 | 1.53 | 66 | 2.88 | 0.68 | 1.52 | 4.25 | 187 | 1.71 | 0.25 | 1.21 | 2.21 |
|  | **Total** | Female | 144 | 0.98 | 0.27 | 0.42 | 1.53 | 108 | 1.55 | 0.47 | 0.60 | 2.49 | 252 | 1.14 | 0.24 | 0.65 | 1.63 |
|  |  | Male | 135 | 0.91 | 0.18 | 0.55 | 1.27 | 81 | 1.62 | 0.30 | 1.01 | 2.23 | 216 | 1.12 | 0.17 | 0.78 | 1.46 |
|  |  | Total | 279 | 0.94 | 0.21 | 0.52 | 1.36 | 189 | 1.59 | 0.29 | 0.99 | 2.18 | 468 | 1.13 | 0.18 | 0.77 | 1.49 |
| Preschooler | **48 - 59 months** | Female | 69 | 3.96 | 0.80 | 2.35 | 5.57 | 40 | 5.19 | 0.45 | 4.27 | 6.10 | 109 | 4.32 | 0.57 | 3.18 | 5.46 |
|  |  | Male | 69 | 4.25 | 0.55 | 3.14 | 5.36 | 49 | 5.85 | 0.47 | 4.90 | 6.79 | 118 | 4.72 | 0.42 | 3.88 | 5.56 |
|  |  | Total | 138 | 4.11 | 0.60 | 2.91 | 5.31 | 89 | 5.53 | 0.41 | 4.70 | 6.35 | 227 | 4.53 | 0.43 | 3.67 | 5.39 |
|  | **60 - 71 months** | Female | 70 | 5.10 | 0.65 | 3.79 | 6.41 | 36 | 7.08 | 0.93 | 5.21 | 8.96 | 106 | 5.68 | 0.54 | 4.59 | 6.77 |
|  |  | Male | 76 | 6.58 | 0.77 | 5.03 | 8.14 | 32 | 6.04 | 0.80 | 4.44 | 7.65 | 108 | 6.43 | 0.60 | 5.22 | 7.63 |
|  |  | Total | 146 | 5.86 | 0.45 | 4.96 | 6.76 | 68 | 6.55 | 0.73 | 5.08 | 8.02 | 214 | 6.06 | 0.37 | 5.32 | 6.81 |
|  | **Total** | Female | 139 | 4.53 | 0.67 | 3.17 | 5.88 | 76 | 6.13 | 0.50 | 5.12 | 7.13 | 215 | 4.99 | 0.49 | 4.01 | 5.98 |
|  |  | Male | 145 | 5.40 | 0.49 | 4.42 | 6.39 | 81 | 5.94 | 0.40 | 5.13 | 6.75 | 226 | 5.56 | 0.36 | 4.83 | 6.29 |
|  |  | Total | 284 | 4.98 | 0.45 | 4.08 | 5.88 | 157 | 6.03 | 0.31 | 5.42 | 6.65 | 441 | 5.29 | 0.32 | 4.65 | 5.92 |
| **Total** | | Female | 283 | 2.33 | 0.47 | 1.40 | 3.27 | 184 | 3.29 | 0.58 | 2.12 | 4.46 | 467 | 2.61 | 0.37 | 1.86 | 3.36 |
|  |  | Male | 280 | 2.63 | 0.47 | 1.68 | 3.57 | 162 | 3.26 | 0.41 | 2.44 | 4.08 | 442 | 2.81 | 0.36 | 2.08 | 3.54 |
|  |  | Total | 563 | 2.48 | 0.41 | 1.66 | 3.31 | 346 | 3.27 | 0.30 | 2.66 | 3.89 | 909 | 2.72 | 0.31 | 2.10 | 3.34 |
| SE: Standard Error, CI: Confidence Interval  * Weighting was used to adjust the relative contribution of the respondents using known populational characteristics including age, gender, and location of residence according to Iran's latest population census in 2016. | | | | | | | | | | | | | | | | | |
